# Liver Enzymes and Risk of Ischemic Heart Disease and Type 2 Diabetes Mellitus: A Mendelian Randomization Study

**DOI:** 10.1038/srep38813

**Published:** 2016-12-20

**Authors:** Junxi Liu, Shiu Lun Au Yeung, Shi Lin Lin, Gabriel M. Leung, C. Mary Schooling

**Affiliations:** 1 School of Public Health, Li Ka Shing Faculty of Medicine, The University of Hong Kong, Hong Kong SAR, China; 2 City University of New York Graduate School of Public Health and Health Policy, New York, NY, USA

## Abstract

We used Mendelian randomization to estimate the causal effects of the liver enzymes, alanine aminotransferase (ALT), alkaline phosphatase (ALP) and gamma glutamyltransferase (GGT), on diabetes and cardiovascular disease, using genetic variants predicting these liver enzymes at genome wide significance applied to extensively genotyped case-control studies of diabetes (DIAGRAM) and coronary artery disease (CAD)/myocardial infarction (MI) (CARDIoGRAMplusC4D 1000 Genomes). Genetically higher ALT was associated with higher risk of diabetes, odds ratio (OR) 2.99 per 100% change in concentration (95% confidence interval (CI) 1.62 to 5.52) but ALP OR 0.92 (95% CI 0.71 to 1.19) and GGT OR 0.88 (95% CI 0.75 to 1.04) were not. Genetically predicted ALT, ALP and GGT were not clearly associated with CAD/MI (ALT OR 0.74, 95% CI 0.54 to 1.01, ALP OR 0.86, 95% CI 0.64 to 1.16 and GGT OR 1.08, 95% CI 0.97 to 1.19). We confirm observations of ALT increasing the risk of diabetes, but cannot exclude the possibility that higher ALT may protect against CAD/MI. We also cannot exclude the possibility that GGT increases the risk of CAD/MI and reduces the risk of diabetes. Informative explanations for these potentially contradictory associations should be sought.

Observational studies usually show some measures of liver function, such as alanine aminotransferase (ALT), alkaline phosphatase (ALP) and gamma glutamyltransferase (GGT), associated with higher risk of cardiovascular disease (CVD) and type 2 diabetes mellitus (T2DM). Among these liver enzymes, gamma glutamyltransferase (GGT) is most strongly positively associated with both CVD^1^,^2^ and T2DM^3^,^4^, although GGT is a non-specific marker of liver function. Alanine aminotransferase (ALT) is more clearly positively associated with T2DM^4^,^5^ than with CVD^6^,^7^ while the role of alkaline phosphatase (ALP)^8^,^9^ is unclear. Apart from the difficulties of separating out the roles of these correlated liver enzymes, observational studies are open to unmeasured confounding by factors such as alcohol use, pre-existing disease, lifestyle and socioeconomic position, making it uncertain whether liver function could be a valid target of intervention or is even etiologically relevant to these major complex chronic diseases.

In this situation comparing the risk of CVD and T2DM by genetically determined liver function, i.e., Mendelian randomization (MR), provides a way forward. MR takes advantage of the random allocation of genetic endowment at conception to provide randomization analogous to the randomization in randomized controlled trials^10^,^11^ and is an increasingly popular means of obtaining un-confounded estimates. No previous MR study has examined the role of liver enzymes in CVD and T2DM. To clarify their roles, we assessed the association of genetically predicted liver enzymes (ALT, ALP and GGT) with ischemic heart disease (IHD) using large extensively genotyped case-control studies of coronary artery disease (CAD)/myocardial infarction (MI) and T2DM^12^,^13^,^14^,^15^. Given the role of the liver in lipid and glucose metabolism, we also similarly assessed the associations of these liver enzymes with lipids and glucose metabolism using and large extensively genotyped cross-sectional studies of lipids^16^ and glycemic traits^17^.

## Results

At genome wide significance GWAS gave 4 SNPs independently predicting ALT, 14 SNPs independently predicting ALP and 26 SNPs independently predicting GGT^18^. One SNP, rs2954021 (*TRIB1*), predicted both ALT and ALP, meaning that it is pleiotropic. Supplementary Table 1 gives the information extracted for each SNP for CAD/MI and T2DM.

### Genetic associations with CAD/MI

Genetically predicted ALT was not clearly associated with CAD/MI using IVW and all SNPs (see Supplementary Fig. S1b), after excluding potentially pleiotropic SNPs or using more conservative methods, although all the estimates for ALT were in the direction of lower risk but with confidence intervals including the null value (Table 1). Genetically predicted ALP was inversely associated with CAD/MI using IVW (see Supplementary Fig. S2b) or any other method, but this association was not robust to exclusion of potentially pleiotropic SNPs with the exclusion of rs579459 in *ABO* contributing most to the difference. Genetically predicted GGT was not clearly associated with CAD/MI using IVW (see Supplementary Fig. S3b) or any other method, although the direction was towards higher risk but the confidence intervals included the null value. There was no evidence that the MR-Egger intercepts differed from the null for the associations of ALT, ALP or GGT with CAD/MI, particularly after excluding potentially pleiotropic SNPs (Table 1).

### Genetic associations with T2DM

Genetically predicted ALT was positively associated with T2DM using IVW with directionally similar estimates for most SNPs (see Supplementary Fig. S1a). The estimate was very similar after excluding potentially pleiotropic SNPs but this association was not robust to the MR-Egger method which gave much less weight to the SNP (rs2954021) from *PNPLA3*. Genetically predicted ALP was not clearly associated with T2DM using IVW (see Supplementary Fig. S2a). Genetically predicted GGT was inversely associated with T2DM using IVW (see Supplementary Fig. S3a), and the estimates were directionally similar using other methodological approaches, but the confidence intervals included the null value. There was no evidence that the MR-Egger intercepts differed from the null for the associations of ALT, ALP or GGT with T2DM, particularly after excluding potentially pleiotropic SNPs (Table 1).

### Genetic associations with lipids and glycemic traits

Genetically predicted ALT, ALP and GGT tended to be inversely related to both LDL- and HDL- cholesterol (Table 2). Among people without diabetes, genetically predicted ALT, ALP and GGT tended to have associations with measures of glucose metabolism directionally consistent with the respective estimates for T2DM, but most confidence intervals included the null value (Table 3). There was no evidence that the MR-Egger intercepts differed from the null for the associations of ALT, ALP or GGT with lipids or glycemic traits (Tables 2 and 3).

## Discussion

This novel study is consistent with most previous observational studies showing higher ALT associated with a higher risk of T2DM^4^,^19^. Our findings are also consistent with observed positive associations of GGT with ischemic heart disease (IHD)^1^,^2^. Our study is also suggestive of an inverse association of ALT with IHD, and of GGT with T2DM. As such, this study considering each liver enzyme independently has confirmed some previous observations but raised questions about the role of ALT in IHD and of GGT in diabetes which may previously have been obscured by correlations between markers of liver function.

MR provides a means of obtaining un-confounded estimates, because genetic make-up is randomly allocated at conception and so is unlikely to be influenced by confounders, such as lifestyle, heath status or socioeconomic position. The risk of chance associations generated by the underlying data structure is reduced by using separate samples for liver enzymes and the outcomes^20^, which is unlikely to be negated by the 5–6% overlap between the samples used to obtain genetic association with the exposures and with the outcomes. All the studies are largely of people of European descent with genomic control^12^,^14^,^15^,^16^,^17^,^18^,^21^,^22^,^23^ which reduces bias from hidden genetic relations. We used SNPs to predict liver enzymes which were from GWAS and were strongly associated with liver enzymes to reduce the risk of false positives. We also checked whether the SNPs used to predict liver enzymes could be associated with the outcomes directly rather than via liver enzymes and repeated the analysis with those potentially pleiotropic SNPs excluded. Nevertheless despite checking the assumptions of Mendelian randomization rigorously, limitations exist. First, given the use of separate samples we could not test whether the associations of liver enzymes with the outcomes vary by level of liver enzymes, by age or by sex, although causal relations are usually consistent. Second, we cannot be certain that the SNPs do not have unknown direct effects on IHD and T2DM. We excluded SNPs with known pleiotropic effects including the SNP (rs2954021) that predicted both ALT and ALP and the estimates were similar for GGT and ALT but less so for ALP, because of the role of rs579459 from the *ABO* gene. Third, estimates may be sensitive to analytic choices, but were generally similar, using weighted median estimates, although the MR-Egger estimates had much wider confidence intervals but gives consistent estimates in the unlikely event of all SNPs being invalid but satisfying the InSIDE assumption^24^. Although the exact functionality of all the SNPs used to predict liver enzymes is not entirely clearly, some of them are expressed in the liver, for example all the 4 SNPs (rs738409 (*PNPLA3*), rs2954021 (*TRIB1*), rs6834314 (*MAPK10, HSD17B1*) and rs10883437 (*CPN1*)) related to ALT are expressed mainly in liver according to data in the Human Protein Altas (http://www.proteinatlas.org/)^25^,^26^, making a causal role plausible and making MR-Egger estimates very conservative^24^. Fourth, canalization, i.e., compensatory mechanisms that drive some of the association of genetic variants with liver enzymes, might result in different associations in MR than would be obtained from interventions changing liver enzymes. However, whether such canalization exists is unknown. Fifth, GGT, ALT and ALP are not only markers of liver disease but are also affected by bone diseases (Paget disease, osteomalacia, rickets), primary and secondary hyperparathyroidism, kidney and pancreatic dysfunction (GGT is primarily present in these cells) and drug use (phenobarbital and phenytoin), so although the estimates represent the effects of each specific liver enzyme they may not only represent liver function^27^,^28^.

The findings for these liver enzymes concerning T2DM show some consistency with observational studies, where ALT is usually positively associated with T2DM^4^,^5^ and has been found associated with death from diabetes related causes^29^. ALT is thought to cause diabetes via insulin resistance^30^ with hepatic steatosis aggravating insulin resistance and creating a vicious cycle^31^. Consistent with this hypothesis genetically predicted ALT also showed indications of a positive association with insulin resistance (Table 3). However, the reason for ALT causing insulin resistance remains elusive. Observationally, ALP is not clearly associated with T2DM^11^,^32^, consistent with these results. Observationally, GGT is also usually positively associated with diabetes^3^,^4^, even using methods that enable correlated exposures, such as liver enzymes to be disentangled^4^. However, our analysis suggests the association for genetically predicted GGT might be in the other direction; confirmation of this result is required.

The findings for the associations of genetically predicted ALT and GGT with IHD are somewhat consistent with observational studies. GGT is often positively associated with IHD^1^,^2^, and our findings are consistent with this interpretation, although the confidence intervals included the null value. ALT usually has a neutral association with IHD^6^, our findings are consistent with a neutral association but cannot rule out an inverse association. The findings for the association of genetically predicted ALP with IHD are difficult to interpret because the negative association depends on rs579459 (near *ABO*) when the reasons for blood groups being associated with IHD are not currently fully understood. It is not clear whether rs579459 is operating via alterations in liver function, is directly functionally relevant to IHD by some yet to be identified mechanism or is a correlate of other factors directly causing IHD.

Overall, these findings indicate complex relations of liver enzymes with IHD and diabetes that may be directionally different even though diabetes is a strong risk factor for IHD. However, it has recently been discovered that key causal factors for IHD may have directionally different relations with IHD and diabetes, such as LDL cholesterol or statins^33^,^34^, which clearly has important implications for prevention and treatment of both conditions. No accepted mechanistic explanation for these paradoxical relation exists, a mechanism via LDL receptor-mediated transmembrane cholesterol transport has been suggested^35^, which is plausible but does not clearly relate to liver function. We have previously suggested a mechanism via sex hormones^36^. Sex hormone receptors are expressed in the liver^37^ and the liver is an important site for sex hormone metabolism^38^ and catabolism^39^,^40^. Randomized controlled trials have shown that estrogen reduces the risk of diabetes^41^ and testosterone improves glucose metabolism^42^,^43^; regulators have warned of the cardiovascular risk of testosterone^44^. However, such an explanation might not explain the different effects of statins, because uncertainties remain as to whether statins affect the liver or cause liver injury or dysfunction^45^, although statins lower sex hormones^46^.

This novel Mendelian randomization study has confirmed some observations concerning poorer liver function, such as ALT likely causing diabetes, but has also raised the possibility of complex effects on IHD. Liver function has complex enzyme and disease specific effects on major non-communicable diseases. Greater understanding of the underlying etiology is needed. As such whether intervening on liver function would improve diabetes without affecting its major consequence, i.e., cardiovascular disease, is unclear. This study also shows the importance of using genetic evidence to identify and select targets of intervention, but leaves several unanswered questions concerning the role of liver function in diabetes and cardiovascular disease. Further investigation is required.

## Methods

### Genetically predicted liver enzymes

Single nucleotide polymorphisms (SNPs) strongly associated with ALT, ALP and GGT at genome wide significance (p-value < 5 × 10^−8^) were obtained from genome wide association studies (GWAS). Any highly correlated SNPs (in linkage disequilibrium) (r^2^ ≥ 0.8) were discarded to retain SNPs with a smaller p-value and/or larger effect size. SNP Annotation and Proxy Search (http://www.broad.mit.edu/mpg/snap/ldsearchpw.php) was used to ascertain these correlations (linkage disequilibrium) using the same catalog as the relevant GWAS. Whether any of the selected SNPs were related to CAD/MI or T2DM directly rather than through liver enzymes (pleiotropic effects) was assessed from their known traits/phenotypes obtained from a comprehensive genotype to phenotype cross-reference, Ensembl (http://www.ensembl.org/index.html).

### Genetically predicted CAD/MI, diabetes, lipids and glycemic traits

Data on CAD/MI have been contributed by CARDIoGRAMplusC4D investigators and have been downloaded from www.CARDIOGRAMPLUSC4D.ORG. CARDIoGRAMplusC4D provides two large overlapping case-control studies largely in people of European descent, one genotyped using 1000 Genomes (60,801 CAD cases, 123,504 controls) and the other genotyped using Hapmap with limited genotyping (63,746 CAD/MI, 130,681 controls) but with more extensive genotyping for a subset (22,233 CAD/MI cases, 64,762 controls)^12^,^14^,^22^. Genetic associations with T2DM are from an extensively genotyped case (n = 34,840)-control (n = 114,981) study largely of people of European descent from the DIAbetes Genetics Replication and Meta-analysis consortium, http://diagramconsortium.org/index.html.^15^ Genetic associations with lipids (inverse normal transformed effect sizes) are from the Global Lipids Genetic Consortium Results of 188,577 people mainly of European ancestry http://csg.sph.umich.edu//abecasis/public/lipids2013/ including low-density lipoprotein (LDL)- cholesterol, high-density lipoprotein (HDL)-cholesterol and triglycerides^16^. Data on glycemics glycemic traits, including glycated hemoglobin (HbA_1_c) (%) (n = 46,368)^23^, fasting glucose (FG) (mmol/L) (n = 122,743)^17^, log transformed β-cell function (HOMA-B) (n = 98,372)^17^, and insulin resistance (HOMA-IR) (n = 98,372)^17^, are in people of European descent without diabetes, and have been contributed by MAGIC investigators and have been downloaded from www.magicinvestigators.org.

### Statistical analyses

Un-confounded estimates of the association of each liver enzyme with CAD/MI, T2DM, lipids and glycemic traits were obtained from separate sample instrumental variable analysis by combining SNP-specific Wald estimates^47^, with the standard error approximated using Fieller’s theorem^48^, using inverse variance weighting (IVW) with fixed effects^49^. The Wald estimate is the ratio of the estimate of SNP on outcome to SNP on liver enzyme.

### Sensitivity analyses

We conducted two sensitivity analyses to assess whether the estimates were robust to methodological choices. First, we repeated the analysis excluding SNPs that might be associated with the relevant outcome directly rather than via liver enzymes, i.e., pleiotropic effects which might violate the exclusion-restriction assumption of instrumental variable analysis. Second, when each SNP contributed less than 50% of the weight, we used a weighted median estimate which may generate correct estimates even when 50% of the SNPs included violate the instrumental variable assumptions^50^. When a single SNP contributed more than 50% we used MR-Egger regression because it may generate correct estimates even when all the SNPs are invalid instruments as long as the instrument strength independent of direct effect (InSIDE) assumption is satisfied^24^. We also examined the value of the intercept term from the MR-Egger regression which gives the average directional pleiotropic effect across genetic variants, i.e., the average direct effect of a variant on the outcome. A p-value of <0.05 indicates the presence of directional pleiotropy across the genetic variants included in the analysis^50^. MR-Egger has a lower false positive rate than IVW but a higher false negative rate^24^.

All statistical analyses were conducted using Stata version 13.1 (StataCorp LP, College Station, TX) and R version 3.3.0 (R Foundation for Statistical Computing, Vienna, Austria). Ethical approval from an Institutional Review Board is not required, since this study only uses publicly available data.

## Additional Information

**How to cite this article**: Liu, J. *et al*. Liver Enzymes and Risk of Ischemic Heart Disease and Type 2 Diabetes Mellitus: A Mendelian Randomization Study. *Sci. Rep.* **6**, 38813; doi: 10.1038/srep38813 (2016).

**Publisher's note:** Springer Nature remains neutral with regard to jurisdictional claims in published maps and institutional affiliations.

## Supplementary Material

Supplementary Information

## Figures and Tables

**Table 1:** Estimates of the effect of genetically predicted liver enzymes ALT, ALP and GGT (per 100% change in concentration)^18^ on coronary artery disease (CAD)/myocardial infarction (MI)^12^,^14^,^22^ and type 2 diabetes mellitus (T2DM)^15^ using Mendelian randomization with different methodological approaches with and without potentially pleiotropic SNPs.

Outcome	data source	Liver Enzyme	All SNPs						Excluding potentially pleiotropic SNPs‡					
			SNPs	Method	OR	95% CI	MR-Egger§		SNPs	Method	OR	95% CI	MR-Egger§	
							Intercept	Intercept p value					Intercept	Intercept p value
CAD/MI	CARDIoGRAMplusC4D	ALT	4	IVW	0.89	0.54 to 1.46	0.07	0.12	3	IVW	0.79	0.48 to 1.31	0.03	0.47
	CARDIoGRAMplusC4D 1000 genomes			IVW	0.79	0.58 to 1.08	0.04	0.28		IVW	0.74	0.54 to 1.01	0.004	0.836
	CARDIoGRAMplusC4D			MR-Egger	0.18	0.01 to 5.75	—	—		MR-Egger	0.43	0.13 to 1.43	—	—
	CARDIoGRAMplusC4D 1000 genomes			MR-Egger	0.33	0.01 to 9.62	—	—		MR-Egger	0.68	0.10 to 4.73	—	—
	CARDIoGRAMplusC4D	ALP	14	IVW	0.72	0.57 to 0.91	0.03	0.06	9	IVW	1.44	0.95 to 2.17	−0.03	0.16
	CARDIoGRAMplusC4D 1000 genomes			IVW	0.61	0.50 to 0.74	0.02	0.08		IVW	0.86	0.64 to 1.16	−0.01	0.72
	CARDIoGRAMplusC4D			MR-Egger	0.38	0.17 to 0.86	—	—		WM	1.54	0.91 to 2.63	—	—
	CARDIoGRAMplusC4D 1000 genomes			WM	0.46	0.35 to 0.60	—	—		WM	0.94	0.60 to 1.48	—	—
	CARDIoGRAMplusC4D	GGT	26	IVW	1.11	0.97 to 1.27	0.004	0.662	23	IVW	1.12	0.97 to 1.29	0.001	0.91
	CARDIoGRAMplusC4D 1000 genomes			IVW	1.01	0.92 to 1.10	0.01	0.34		IVW	1.08	0.97 to 1.19	0.01	0.55
	CARDIoGRAMplusC4D			WM	1.16	0.94 to 1.42	—	—		WM	1.16	0.94 to 1.44	—	—
	CARDIoGRAMplusC4D 1000 genomes			WM	1.03	0.89 to 1.19	—	—		WM	1.05	0.91 to 1.23	—	—
Diabetes	DIAGRAM	ALT	4	IVW	2.68	1.48 to 4.86	−0.01	0.70	3	IVW	2.99	1.62 to 5.52	0.02	0.64
				MR-Egger	3.54	0.17 to 73.79	—	—		MR-Egger	1.99	0.16 to 24.36	—	—
	DIAGRAM	ALP	14	IVW	0.91	0.70 to 1.18	0.01	0.28	13	IVW	0.92	0.71 to 1.19	0.01	0.24
				MR-Egger	0.71	0.35 to 1.45	—	—		MR-Egger	0.68	0.32 to 1.44	—	—
	DIAGRAM	GGT	26	IVW	0.83	0.71 to 0.97	−0.02	0.09	24	IVW	0.88	0.75 to 1.04	−0.01	0.33
				WM	0.82	0.62 to 1.10	—	—		WM	0.91	0.68 to 1.21	—	—

IVW: Inverse Variance Weighted, WM: Weighted Median.

^‡^CAD/MI related SNPs excluded for ALT: rs2954021 (*TRIB1*), excluded for ALP: rs174601 (*C11orf10, FADS1, FADS2*), rs314253 (*ASGR1, DLG4*), rs2954021 (*TRIB1*), rs579459 (*ABO*) and rs6984305 (*PPP1R3B*), excluded for GGT: rs516246 (*FUT2*), rs7310409 (*HNF1A*) and rs1260326 (*C2orf16, GCKR*).

Diabetes related SNPs excluded for GGT: rs516246 (*FUT2*) and rs1260326 (*C2orf16, GCKR*); rs2954021 (*TRIB1*) excluded for ALT and ALP.

^§^The intercept can be interpreted as an estimate of the average pleiotropic effect across the genetic variants where a corresponding p-value of <0.05 indicates the presence of directional pleiotropy across the genetic variants included in the analyses.

**Table 2:** Estimates of the effects of genetically predicted liver enzymes ALT, ALP and GGT (per 100% change in concentration)^18^ on lipids^16^ using Mendelian randomization with different methodological approaches with and without potentially pleiotropic SNPs.

Liver enzyme	Lipid	All SNPs						Excluding potentially pleiotropic SNPs related to lipids‡					
		SNPs	Method	Beta	95%CI	MR-Egger§		SNPs	Method	Beta	95%CI	MR-Egger§	
						Intercept	Intercept p value					Intercept	Intercept p value
ALT	LDL cholesterol (SD)	4	IVW	−0.19	−0.37 to −0.01	0.05	0.3	3	IVW	−0.21	−0.39 to −0.03	0.003	0.861
			MR-Egger	−1.20	−5.68 to 3.28	—	—		MR-Egger	−0.29	−5.25 to 4.66	—	—
	HDL cholesterol (SD)	4	IVW	−0.22	−0.39 to −0.06	−0.03	0.43	3	IVW	−0.20	−0.37 to −0.03	0.003	0.908
			MR-Egger	0.30	−3.11 to 3.72	—	—		MR-Egger	−0.30	−9.01 to 8.40	—	—
	Triglycerides (SD)	4	IVW	−0.10	−0.17 to 0.15	0.04	0.48	3	IVW	−0.03	−0.18 to 0.13	−0.02	0.62
			MR-Egger	−0.81	−6.70 to 5.08	—	—		MR-Egger	0.36	−8.34 to 9.06	—	—
ALP	LDL cholesterol (SD)	14	IVW	−0.47	−0.57 to −0.36	0.01	0.65	9	IVW	−0.08	−0.23 to 0.08	−0.01	0.57
			WM	−0.71	−0.94 to −0.49	—	—		WM	−0.11	−0.33 to 0.10	—	—
	HDL cholesterol (SD)	14	IVW	−0.09	−0.17 to −0.01	−0.01	0.48	9	IVW	0.11	−0.04 to 0.25	0.01	0.53
			MR-Egger	−0.05	−0.75 to 0.66	—	—		WM	−0.03	−0.23 to 0.18	—	—
	Triglycerides (SD)	14	IVW	0.50	−0.03 to 0.13	0.01	0.67	9	IVW	−0.19	−0.33 to −0.04	−0.01	0.3
			MR-Egger	0.02	−0.77 to 0.81	—	—		WM	−0.01	−0.21 to 0.19	—	—
GGT	LDL cholesterol (SD)	26	IVW	−0.05	−0.10 to −0.01	0.01	0.06	23	IVW	−0.04	−0.09 to 0.02	−0.0002	0.9511
			WM	−0.06	−0.14 to 0.02	—	—		WM	−0.05	−0.14 to 0.03	—	—
	HDL cholesterol (SD)	26	IVW	−0.07	−0.11 to −0.03	0.002	0.588	23	IVW	−0.03	−0.08 to 0.01	0.003	0.430
			WM	−0.07	−0.14 to −0.01	—	—		WM	−0.04	−0.11 to 0.02	—	—
	Triglycerides (SD)	26	IVW	0.03	−0.01 to 0.07	0.02	0.21	23	IVW	0.01	−0.04 to 0.06	0.002	0.842
			WM	−0.02	−0.08 to 0.04	—	—		WM	−0.04	−0.11 to 0.04	—	—

IVW: Inverse Variance Weighted; WM: Weighted Median.

^‡^Lipids related SNPs excluded for ALT: rs2954021 (*TRIB1*), excluded for ALP: rs174601 (*C11orf10, FADS1, FADS2*), rs314253 (*ASGR1, DLG4*), rs2954021 (*TRIB1*), rs579459 (*ABO*) and rs6984305 (*PPP1R3B*), excluded for GGT: rs516246 (*FUT2*), rs7310409 (*HNF1A*) and rs1260326 (*C2orf16, GCKR*).

^§^The intercept can be interpreted as an estimate of the average pleiotropic effect across the genetic variants where a corresponding p-value of <0.05 indicates the presence of directional pleiotropy across the genetic variants included in the analyses.

**Table 3:** Estimates of the effects of genetically predicted liver enzymes ALT, ALP and GGT (per 100% changes in concentration)^18^ on glycemic traits^17^,^23^ using Mendelian randomization with different methodological approaches with and without potentially pleiotropic SNPs.

Liver enzyme	Glycemic Traits	All SNPs						Excluding potentially pleiotropic SNPs related to obesity or another liver enzymes‡					
		SNPs	Method	Beta	95%CI	MR-Egger§		SNPs	Method	Beta	95%CI	MR-Egger§	
						Intercept	Intercept p value					Intercept	Intercept p value
ALT	HbA1c (%)	4	IVW	0.006	−0.105 to 0.118	−0.003	0.765	3	IVW	0.03	−0.08 to 0.14	0.01	0.42
			MR-Egger	0.07	−1.02 to 1.15	—	—		MR-Egger	−0.14	−2.06 to 1.78	—	—
	Fasting glucose (mmol/L)	4	IVW	0.05	−0.06 to 0.17	−0.005	0.378	3	IVW	0.07	−0.05 to 0.19	−0.004	0.628
			MR-Egger	0.17	−0.07 to 0.41	—	—		MR-Egger	0.15	−0.77 to 1.08	—	—
	Insulin resistance	4	IVW	0.08	−0.04 to 0.21	−0.003	0.546	3	IVW	0.09	−0.04 to 0.22	−0.004	0.639
			MR-Egger	0.17	−0.09 to 0.43	—	—		MR-Egger	0.18	−0.60 to 0.96	—	—
	Beta cell function	4	IVW	−0.01	−0.12 to 0.09	−0.002	0.657	3	IVW	−0.02	−0.13 to 0.09	−0.004	0.552
			MR-Egger	0.03	−0.21 to 0.27	—	—		MR-Egger	0.08	−0.25 to 0.42	—	—
ALP	HbA1c (%)	14	IVW	−0.09	−0.15 to −0.02	0.001	0.636	13	IVW	−0.08	−0.15 to −0.01	0.003	0.222
			MR-Egger	−0.12	−0.30 to 0.05	—	—		MR-Egger	−0.156	−0.312 to 0.001	—	—
	Fasting glucose (mmol/L)	14	IVW	−0.12	−0.19 to −0.04	0.001	0.800	13	IVW	−0.12	−0.19 to −0.04	0.002	0.766
			MR-Egger	−0.14	−0.49 to 0.21	—	—		MR-Egger	−0.15	−0.52 to 0.23	—	—
	Insulin resistance	14	IVW	−0.09	−0.17 to −0.01	−0.001	0.827	13	IVW	−0.09	−0.17 to −0.01	−0.001	0.781
			MR-Egger	−0.06	−0.31 to 0.20	—	—		MR-Egger	−0.05	−0.33 to 0.23	—	—
	Beta cell function	14	IVW	−0.004	−0.068 to 0.061	−0.002	0.537	13	IVW	−0.004	−0.069 to 0.061	−0.002	0.521
			MR-Egger	0.05	−0.17 to 0.27	—	—		MR-Egger	0.06	−0.18 to 0.29	—	—
GGT	HbA1c (%)	26	IVW	−0.01	−0.04 to 0.03	−0.003	0.152	24	IVW	−0.002	−0.035 to 0.032	−0.002	0.279
			WM	0.02	−0.03 to 0.07	—	—		WM	0.02	−0.03 to 0.07	—	—
	Fasting glucose (mmol/L)	26	IVW	−0.01	−0.05 to 0.02	−0.01	0.11	24	IVW	−0.001	−0.038 to 0.036	−0.003	0.253
			WM	0.03	−0.02 to 0.08	—	—		WM	0.03	−0.02 to 0.09	—	—
	Insulin resistance	26	IVW	−0.02	−0.06 to 0.02	−0.003	0.331	24	IVW	−0.01	−0.05 to 0.03	0.00	0.97
			WM	−0.03	−0.08 to 0.03	—	—		WM	−0.03	−0.08 to 0.03	—	—
	Beta cell function	26	IVW	−0.01	−0.04 to 0.02	0.001	0.696	24	IVW	−0.005	−0.038 to 0.028	0.001	0.454
			WM	−0.04	−0.10 to 0.01	—	—		WM	−0.04	−0.08 to 0.01	—	—

IVW: Inverse Variance Weighted; WM: Weighted Median.

^‡^Excluding SNP (rs2954021 (*TRIB1*)) for ALT and ALP, excluding glycemic traits related SNPs for GGT: rs516246 (*FUT2*) and rs1260326 (*C2orf16, GCKR*).

^§^The intercept can be interpreted as an estimate of the average pleiotropic effect across the genetic variants where a corresponding p-value of <0.05 indicates the presence of directional pleiotropy across the genetic variants included in the analyses.
