# Supplementary material for: Liver Enzymes and Risk of Ischemic Heart Disease and Type 2 Diabetes Mellitus: A Mendelian Randomization Study
Source: Sci Rep. 2016 Dec 20;6:38813. doi: 10.1038/srep38813 (PMC5171875; doi:10.1038/srep38813)

Liver Enzymes and Risk of Ischemic Heart Disease and Type 2 Diabetes Mellitus: A Mendelian Randomization Study

Junxi Liu[1], Shiu Lun Au Yeung[1], Shi Lin Lin[1], Gabriel M Leung[1], C Mary Schooling[1,2 *]
[1]School of Public Health, Li Ka Shing Faculty of Medicine, The University of Hong Kong, Hong Kong SAR, China

[2]City University of New York Graduate School of Public Health and Health Policy, New York, NY, USA

*Corresponding author:

Dr. C Mary Schooling

School of Public Health

Li Ka Shing Faculty of Medicine

The University of Hong Kong

G/F, Patrick Manson Building (North Wing), 7 Sassoon Road,

Hong Kong SAR, China

Telephone: (852) 3917 6732

Fax: (852) 3520 1945

E-mail: cms1@hku.hk

Supplementary table 1. Characteristics of SNPs used in the Mendelian randomization analysis of the effect of GGT, ALP and ALT on the risk of coronary artery disease (CAD) /myocardial infarction (MI)[12,14,22] and type 2 diabetes mellitus (T2DM)[15]

Obtained from GWAS based on SNPs from Chambers et al., 2011							Obtained from CARDIoGRAMplusC4D 1000 Genomes-based		Obtained from CARDioGRAM Metabochip			Obtained from DIAGRAM		
Enzyme	Rs Code	Gene	Effect Allele	Other Allele	Increase in exposure	P value	Increase in log odds per effect allele	Log standard error of gene-CAD relation	Obtain from	Increase in log odds per effect allele	Log standard error of gene-CAD relation	Obtain from	Increase in log odds per effect allele	Log standard error of gene-T2DM relation
ALT	rs738409	*PNPLA3*	G	C	6	1.20E-45	0.019307	0.0110051	CARDIoGRAM	0.02392	0.017783	DIAGRAM 2012	0.0582689	0.0215708
ALT	rs2954021	*TRIB1*	A	G	1.6	5.30E-09	-0.044263	0.0091943	CARDIoGRAM	-0.06237	0.01391	DIAGRAM 2013	-0.0143018	0.0201519
ALT	rs6834314	*MAPK10, HSD17B13*	A	G	2.6	3.10E-09	-0.009664	0.010727	CARDIoGRAM	-0.00443	0.015938	DIAGRAM 2012	0.0392207	0.0196329
ALT	rs10883437	*CPN1*	T	A	2.3	4.00E-09	-0.00243	0.0101056	CARDIoGRAM	0.008571	0.020634	DIAGRAM 2012	0.0295588	0.0198237
ALP	rs174601	*TMEM258, FADS1*	T	C	1.7	2.60E-09	0.0216	0.0104055	CARDIoGRAM	0.0047409	0.0173851	DIAGRAM 2013	-0.0245151	0.0125481
ALP	rs281377	*FUT2*	T	C	1.8	1.10E-15	-0.014042	0.0097917	CARDIoGRAM	0.010772	0.0209767	DIAGRAM 2012	0.0099503	0.0228602
ALP	rs314253	*ASGR1, DL G4*	C	T	2.1	8.40E-12	0.012462	0.0099047	CARDIoGRAM plusC4D	0.011008	0.009375	DIAGRAM 2013	0.0100404	0.0119368
ALP	rs579459	*ABO*	T	C	8.8	2.60E-123	-0.072956	0.0113168	CARDIoGRAM plusC4D	0.071151	0.013195	DIAGRAM 2013	0.0296103	0.0145656
ALP	rs1883415	*ALDH5A1, GPLD1*	C	A	3.1	5.60E-26	-0.006256	0.0098547	CARDIoGRAM	0.0231868	0.0150773	DIAGRAM 2012	0.0198026	0.0174284
ALP	rs1976403	*ALPL, NBPF 3*	C	A	3.6	1.80E-50	0.024152	0.010916	CARDIoGRAM	0.0174892	0.0168706	DIAGRAM 2012	0.0099503	0.0176002
ALP	rs2236653	*ST3GAL4*	T	C	1.5	1.80E-09	-0.007767	0.0094637	CARDIoGRAM	0.0379043	0.0150391	DIAGRAM 2012	0.0099503	0.0176002
ALP	rs2954021	*TRIB1*	A	G	1.4	2.30E-13	-0.044263	0.0091943	CARDIoGRAM	-0.0623655	0.0139097	DIAGRAM 2013	-0.0143018	0.0201519
ALP	rs6984305	*PPP1R3B*	A	T	2.7	2.10E-10	0.010665	0.0151101	CARDIoGRAM plusC4D	0.001348	0.01386	DIAGRAM 2013	0.0688168	0.0184373
ALP	rs7186908	*HPR, PMFB P1*	C	G	2	4.80E-09	-0.019514	0.0107563	CARDIoGRAM	0.0434959	0.0170504	DIAGRAM 2012	0.0487901	0.0219841
ALP	rs7267979	*ABHD12, GI NS1, PYGB*	G	A	1.5	7.40E-10	0.025203	0.0095028	CARDIoGRAM plusC4D	0.006075	0.00875	DIAGRAM 2013	0.00615	0.0115178
ALP	rs7923609	*JMJD1C, NR BF2*	G	A	2.2	5.90E-23	-0.011546	0.0091443	CARDIoGRAM plusC4D	0.008236	0.008564	DIAGRAM 2013	0.0042998	0.0117963
ALP	rs10819937	*ALDOB*	C	G	2.5	1.00E-09	-0.000555	0.0123303	CARDIoGRAM	0.0254407	0.0204888	DIAGRAM 2012	0	0.0255315
ALP	rs16856332	*ABCB11*	T	G	3.9	1.60E-09	-0.010252	0.0236157	CARDIoGRAM	-0.0050296	0.038357	DIAGRAM 2012	0.0198026	0.0474233
GGT	rs339969	*RORA*	A	C	4.5	6.60E-20	-0.004425	0.0097917	CARDIoGRAM	0.0142294	0.0160774	DIAGRAM 2012	0.0198026	0.0200182

GGT	rs516246	*FUT2*	T	C	2.3	7.60E-10	-0.006235	0.0099079	CARDIoGRAM	0.0013034	0.0182692	DIAGRAM 2013	0.0179214	0.0128226
GGT	rs754466	*DLG5*	T	A	3.5	6.40E-10	-0.007444	0.0118089	CARDIoGRAM	0.0250517	0.0174476	DIAGRAM 2012	0.0392207	0.0196329
GGT	rs944002	*EXOC3L4*	G	A	6.3	5.80E-29	-0.028013	0.0113375	CARDIoGRAM	0.0226158	0.0213826	DIAGRAM 2012	0.0099503	0.0276737
GGT	rs1076540	*MICAL3*	C	T	4.8	9.60E-17	0.003257	0.0096201	CARDIoGRAM	-0.0060121	0.0169456	DIAGRAM 2012	0.0198026	0.0226347
GGT	rs1260326	*C2orf16, GCKR*	T	C	3.2	3.90E-13	-0.007116	0.0133286	CARDIoGRAM	-0.0238895	0.0145478	DIAGRAM 2013	-0.0591918	0.0123384
GGT	rs1335645	*CEPT1, DENND2D*	A	G	4.3	7.30E-09	0.027339	0.0095125	CARDIoGRAM	-0.0221269	0.0221847	DIAGRAM 2012	0	0.030649
GGT	rs1497406	*RSG1, EPHA2*	G	A	3.8	2.80E-19	-0.005233	0.0114135	CARDIoGRAM	0.0346128	0.0140945	DIAGRAM 2012	0.0099503	0.0202167
GGT	rs2073398	*GGT1, GGTLC2*	G	C	12.3	1.10E-109	0.014372	0.0098688	CARDIoGRAM	-0.0207129	0.0171149	DIAGRAM 2012	0.0295588	0.0198237
GGT	rs2140773	*EFHD1, LOC100129166*	A	C	2.9	1.10E-09	-0.019816	0.0111181	CARDIoGRAM plusC4D	0.000211	0.009878	DIAGRAM 2013	0.0066399	0.0128368
GGT	rs2739330	*DDT, DDTL*	T	C	3.7	1.70E-09	-0.075164	0.0170057	CARDIoGRAM	-0.0468233	0.0191537	DIAGRAM 2012	0.0295588	0.0198237
GGT	rs4074793	*ITGA1*	G	A	5.5	3.40E-10	-0.004324	0.0115124	CARDIoGRAM	0.0386583	0.0279373	DIAGRAM 2012	0.0295588	0.032084
GGT	rs4503880	*NEDD4L*	T	C	3.6	3.00E-12	0.001628	0.01147	CARDIoGRAM	0.0048262	0.0178362	DIAGRAM 2012	0.0295588	0.0247867
GGT	rs4547811	*ZNF827*	C	T	6.4	2.50E-27	0.009396	0.0105076	CARDIoGRAM	0.0076317	0.0184948	DIAGRAM 2012	0.0295588	0.0247867
GGT	rs4581712	*DYNLRB2*	A	C	3.2	3.10E-09	-0.018694	0.0109681	CARDIoGRAM	-0.0079261	0.0156553	DIAGRAM 2012	0	0.0177755
GGT	rs6888304	*CDH6*	A	G	2.7	1.20E-09	-0.036466	0.0092822	CARDIoGRAM	-0.0052386	0.0163566	DIAGRAM 2012	0	0.0204191
GGT	rs7310409	*HNF1A*	G	A	6.8	7.00E-45	-0.018796	0.0108432	CARDIoGRAM plusC4D	0.038294	0.02468	DIAGRAM 2013	0.0404219	0.0121058
GGT	rs8038465	*CD276*	T	C	2.4	1.40E-09	-0.015651	0.0100263	CARDIoGRAM	-0.0040623	0.0149279	DIAGRAM 2012	0.0198026	0.0174284
GGT	rs9296736	*MLIP*	T	C	3	2.60E-09	0.014962	0.0097037	CARDIoGRAM plusC4D	0.011525	0.010121	DIAGRAM 2013	-0.0029744	0.0125109
GGT	rs9913711	*SOX9*	C	G	2.4	1.30E-09	0.003807	0.0098493	CARDIoGRAM	0.0274274	0.0147245	DIAGRAM 2012	0	0.0177755
GGT	rs10513686	*SLC2A2*	A	G	4.9	6.10E-11	0.022278	0.0136219	CARDIoGRAM plusC4D	0.005334	0.012665	DIAGRAM 2013	-0.0209714	0.0173533
GGT	rs10908458	*DPM3, EFNA1, PKLR*	T	C	3.7	1.70E-15	-0.020858	0.009518	CARDIoGRAM	-0.0095768	0.0142242	DIAGRAM 2012	0.0099503	0.0202167
GGT	rs12145922	*CCBL2, PKN2*	A	C	2.8	3.80E-11	-0.015109	0.0093898	CARDIoGRAM	0.0286854	0.0141106	DIAGRAM 2012	0.0392207	0.0196329
GGT	rs12968116	*ATP8B1*	C	T	4.8	8.90E-10	-0.020571	0.0164473	CARDIoGRAM	-0.019313	0.0214642	DIAGRAM 2012	0.0676587	0.0286396
GGT	rs13030978	*MYO1B, STAT4*	T	C	3.7	1.10E-11	-0.007046	0.0105662	CARDIoGRAM	0.012133	0.0158616	DIAGRAM 2012	0.0392207	0.0196329
GGT	rs17145750	*MLXIPL*	C	T	4.5	2.90E-09	0.006561	0.0139304	CARDIoGRAM plusC4D	0.020061	0.012632	DIAGRAM 2013	0.0352436	0.0167767

Supplementary table 2: Single nucleotide polymorphisms (SNP) with potential pleiotropic effects other than via the specific liver enzyme from Ensembl.

	SNPs	Location	Gene nearby	Phenotype, disease and trait
ALT	rs738409	22q13a	*PNPLA3*	Nonalcoholic fatty liver disease(NFLD)
ALT	rs2954021	8q24	*TRIB1*	HDL, LDL, TC and ALP
ALP	rs174601	11q12	*TMEM258, FADS1, FADS2*	Blood metabolite levels, HDL, Red blood cell fatty acid levels, TC, Trans fatty acid levels
ALP	rs314253	17p13	*ASGR1, DLG4*	TC, LDL
ALP	rs579459	9q34a	*ABO*	Blood Metabolite ratios, Coronary artery disease, Ischemic stroke, Large artery stroke, E-selection, Red blood cell traits, Soluble E-selectin levels, Soluble levels of adhesion molecules, TC, Urinary metabolites
ALP	rs2954021	8q24	*TRIB1*	HDL, LDL, TC and ALT
ALP	rs6984305	8p23	*PPP1R3B*	HDL, TC
ALP	rs7923609	10q21a	*ABHD12, GINS1, PYGB*	Educational attainment
GGT	rs516246	16q23	*FUT2*	Crohn's disease, Obesity-related traits, TC
GGT	rs7310409	12q24[a]	*HNF1A*	C-reactive protein, Pancreatic neoplasms, TC
GGT	rs944002	14q32	*C14orf73*	Mean platelet volume
GGT	rs1260326	2p23	*C2orf16, GCKR*	Blood metabolite levels, C-reactive protein, Cholesterol, TC, Chronic kidney disease, Coffee consumption, Fasting Glucose, Glycemic traits, Gout, Hematological and biochemical traits, Hypertriglyceridemia, Lipid metabolism phenotypes, Lipid traits, Lipoprotein-associated phospholipase A2 activity and mass, Metabolic traits, Metabolite levels, Non-albumin protein levels, Platelet counts, Serum albumin level, Serum total protein level, TC, Triglycerides, Two-hour glucose challenge, Urate levels, Waist circumference and related phenotypes
GGT	rs12968116	2q37	*ATP8B1*	Body Height

Nonalcoholic fatty liver disease (NFLD), low-density lipoprotein (LDL)-cholesterol, high-density lipoprotein (HDL)-cholesterol, total cholesterol (TC)

Supplementary figures 1-3:

Figure-1. Estimates of the effect of genetically predicted ALT (per 100% changes in concentration) on coronary artery disease (CAD) /myocardial infarction (MI) (CARDIoGRAMplusC4D 1000 Genomes-based)[22] and type 2 diabetes mellitus)[15] using Mendelian randomization with inverse variance weighting including potentially pleiotropic SNPs

Figure 1a

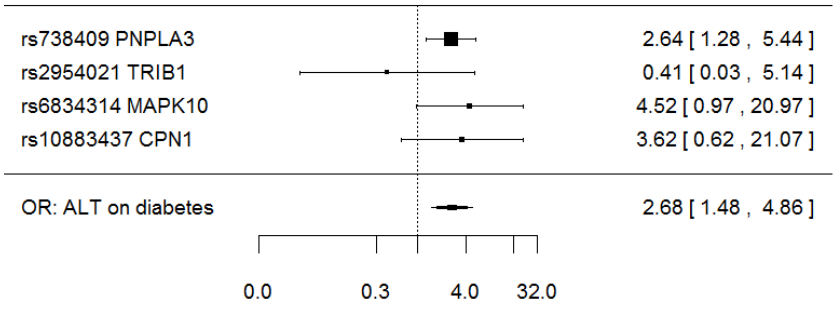

Figure 1b

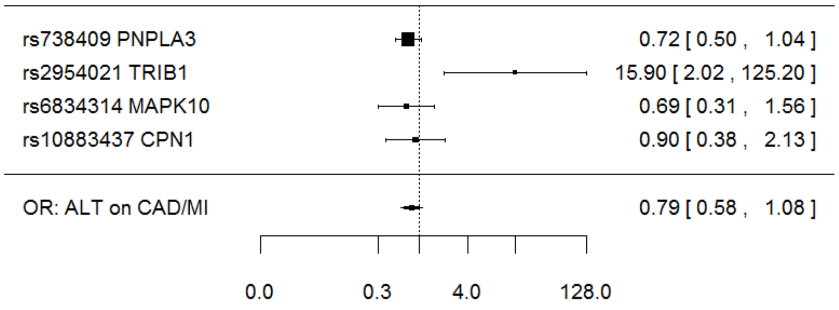

Figure-2. Estimates of the effect of genetically predicted ALP (per 100% changes in concentration) on coronary artery disease (CAD) /myocardial infarction (MI) (CARDIoGRAMplusC4D 1000 Genomes-based)[22] and type 2 diabetes mellitus)[15] using Mendelian randomization with Inverse Variance Weighting including potentially pleiotropic SNPs.

Figure 2a

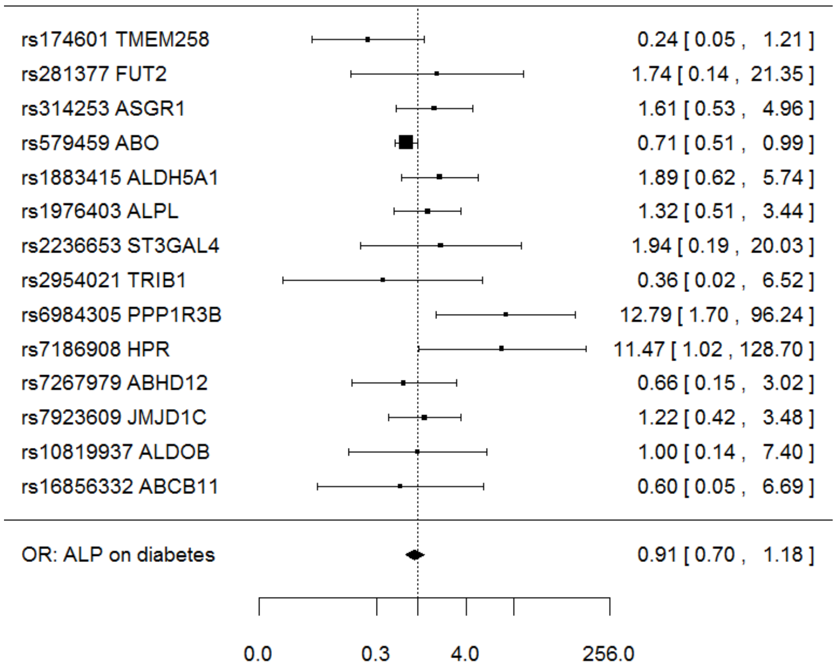

Figure 2b

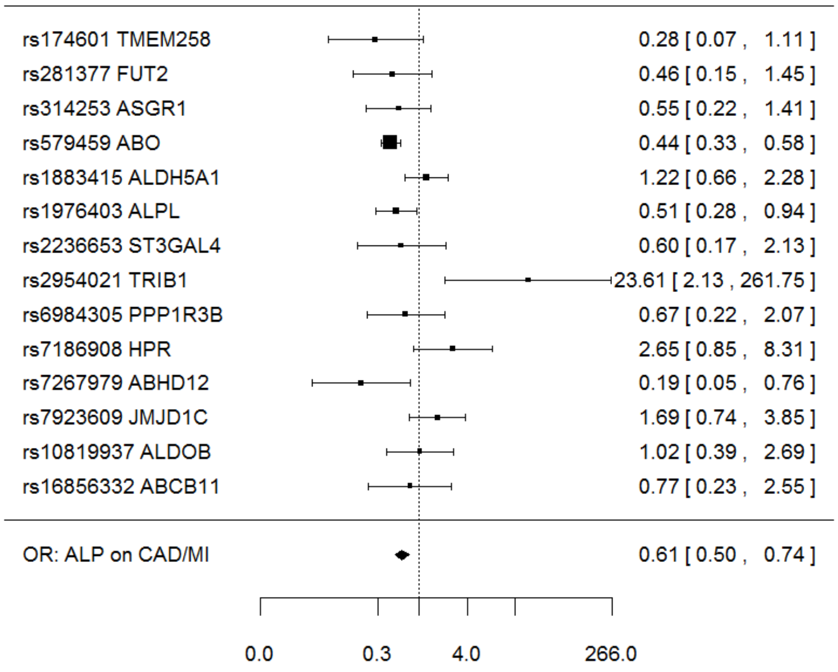

Figure-3. Estimates of the effect of genetically predicted GGT (per 100% changes in concentration) on coronary artery disease (CAD) /myocardial infarction (MI) (CARDIoGRAMplusC4D 1000 Genomes-based)[22] and type 2 diabetes mellitus)[15] using Mendelian randomization with Inverse Variance Weighting including potentially pleiotropic SNPs.

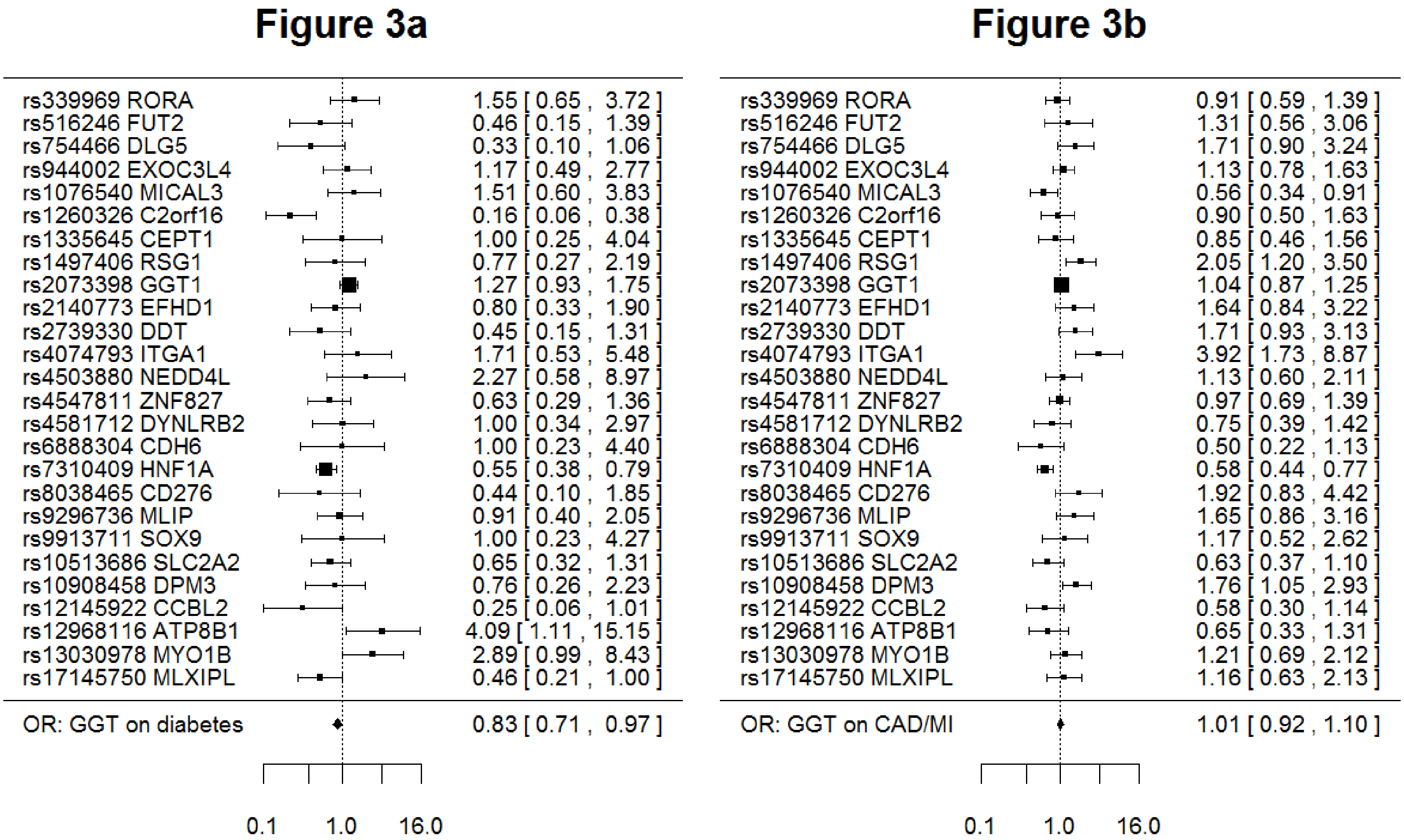

Supplement: Supplementary Information [file srep38813-s1.pdf]
